# Genomics of an extreme psychrophile, *Psychromonas ingrahamii*

**DOI:** 10.1186/1471-2164-9-210

**Published:** 2008-05-06

**Authors:** Monica Riley, James T Staley, Antoine Danchin, Ting Zhang Wang, Thomas S Brettin, Loren J Hauser, Miriam L Land, Linda S Thompson

**Affiliations:** 1 Bay Paul Center, Marine Biological Laboratory, Woods Hole, MA 02543, USA; 2 University of Washington, Seattle, WA 98195-7242, USA; 3 Genetics of Bacterial Genomes, CNRS URA2171, Institut Pasteur, 28 rue du Dr Roux, 75015 Paris, France; 4 DOE Joint Genome Institute, Bioscience Division, Los Alamos National Laboratory, Los Alamos, NM 87545, USA; 5 Oak Ridge National Laboratory, Oak Ridge, TN 37831, USA

## Abstract

**Background:**

The genome sequence of the sea-ice bacterium *Psychromonas ingrahamii *37, which grows exponentially at -12C, may reveal features that help to explain how this extreme psychrophile is able to grow at such low temperatures. Determination of the whole genome sequence allows comparison with genes of other psychrophiles and mesophiles.

**Results:**

Correspondence analysis of the composition of all *P. ingrahamii *proteins showed that (1) there are 6 classes of proteins, at least one more than other bacteria, (2) integral inner membrane proteins are not sharply separated from bulk proteins suggesting that, overall, they may have a lower hydrophobic character, and (3) there is strong opposition between asparagine and the oxygen-sensitive amino acids methionine, arginine, cysteine and histidine and (4) one of the previously unseen clusters of proteins has a high proportion of "orphan" hypothetical proteins, raising the possibility these are cold-specific proteins.

Based on annotation of proteins by sequence similarity, (1) *P. ingrahamii *has a large number (61) of regulators of cyclic GDP, suggesting that this bacterium produces an extracellular polysaccharide that may help sequester water or lower the freezing point in the vicinity of the cell. (2) *P. ingrahamii *has genes for production of the osmolyte, betaine choline, which may balance the osmotic pressure as sea ice freezes. (3) *P. ingrahamii *has a large number (11) of three-subunit TRAP systems that may play an important role in the transport of nutrients into the cell at low temperatures. (4) Chaperones and stress proteins may play a critical role in transforming nascent polypeptides into 3-dimensional configurations that permit low temperature growth. (5) Metabolic properties of *P. ingrahamii *were deduced. Finally, a few small sets of proteins of unknown function which may play a role in psychrophily have been singled out as worthy of future study.

**Conclusion:**

The results of this genomic analysis provide a springboard for further investigations into mechanisms of psychrophily. Focus on the role of asparagine excess in proteins, targeted phenotypic characterizations and gene expression investigations are needed to ascertain if and how the organism regulates various proteins in response to growth at lower temperatures.

## Background

Well over half of the earth's surface is cold: deep oceans, mountains, polar regions. Likewise, Earth's solar system contains many planets and planetary bodies that are also cold. The cold environments on Earth are teeming with life [1] offering hope that other cold environments in our solar system such as Mars and Jupiter's moon, Europa, may harbor life [2]. For this reason it is surprising that so little is know about the lifestyle, particularly of microbial psychrophiles at low temperatures.

Psychrophiles have been studied primarily to understand biological mechanisms of adaptation to extreme conditions. Microbial physiologists have long been interested in psychrophiles as they employ mechanisms allowing them to maintain life processes at temperatures where rates of reactions and molecular properties present challenges. In reaching for an understanding of how life processes work at extremes of temperature, most of the focus to date has been on the properties of enzymes of extremophiles (reviewed by [3,4]). No single consistent answer has emerged to account for adaptation to temperature extremes. To date, no single type of modification is uniformly found in the enzymes of psychrophiles; instead numerous small and subtle differences appear to account for their increased flexibility thereby enabling them to function at low temperatures.

Recently whole genome sequences have been determined for psychrophiles *Colwellia psychrerythraea *34 H [5], *Idiomarina loihiensis *L2TR [6], and *Pseudoalteromonas haloplanktis *TAC125 [7]. We now add the genomic sequence of the extreme species, *Psychromonas ingrahamii *37 which grows at even colder temperatures. Availability of complete genome sequences provides the opportunity to search all of the proteins of the organisms for similarities and differences that might have bearing on the ability of the organism to grow at low temperatures.

The extreme psychrophile, *Psychromonas ingrahamii *was isolated from sea ice from the Arctic. It grows exponentially with a doubling time of 240 hours at -12°C and may well grow at even lower temperatures [8]. These temperatures do not necessarily solidify salt water or cytoplasm into ice. Liquid water has been shown to exist at grain contacts as low as -20C [2].

## Results and Discussion

### The *P. ingrahamii *genome

The single, circular chromosome of 4.56 Mb constituting the genome of *P. ingrahamii *37 was sequenced as a set of contigs by the DOE Joint Genome Institute Production Genomics Facility, 2800 Mitchell Drive, Walnut Creek, CA 94598, and finished at DOE Joint Genome Institute, Bioscience Division, Los Alamos National Laboratory, Los Alamos, NM 87545. It was annotated at Oak Ridge National Laboratory, Oak Ridge, TN 37831 and deposited as GenBank file CP000510.1. Altogether 3708 genes were identified, 3545 of which were proteins of 83 residues or longer. A second round of annotation is described below.

### Properties of proteins

#### Size

**Figure 1:**
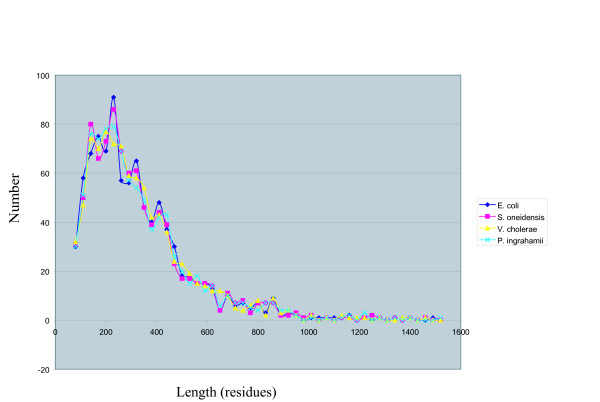
**Distribution of lengths of 916 orthologous proteins in four bacteria**. Distribution of lengths of proteins as numbers of amino acid residues, ranging from 83 to 1501, in increments of 30. Black diamond = *E. coli*, red square = *S. oneidensis*, yellow triangle = *V. cholerae*, blue cross = *P. ingrahamii*.

One can ask whether the sizes of proteins of a psychrophile differ from those of a mesophile. The sequences of all proteins of *P. ingrahamii *were compared to sequences of all proteins of three other bacteria: *Shewanella oneidensis *MR-1, *Vibrio cholerae *and *Escherichia coli *K-12 MG1655. 916 protein sequences were conserved among all four bacteria. The great majority of the conserved proteins were enzymes. The distribution of lengths of the 916 orthologous proteins were compared, revealing that the distribution was about the same for the comparable proteins in all four bacteria (Figure 1). Ideas that proteins that are required to function at low temperatures would be either shorter or longer than those of mesophiles are not borne out.

#### Amino acid composition

Amino acid composition of an organism's proteins is affected by nucleotide composition of the DNA. The GC content of *P. ingrahamii *DNA was determined experimentally to be 40% [8], verified by the composition of the total genome nucleotide sequence (40.1%). Overall amino acid content of the encoded proteins compared to those of *V. cholerae, S. oneidensis *and *E. coli *is shown in Table 1 (P. Sharp, personal comunication). The amino acids isoleucine, asparagine, lysine, phenylalanine and tyrosine all are present in higher percentage in *P. ingrahamii*.

**Table 1:** Amino acid composition (%) of total proteins of 4 bacteria

Organisms*	S	L	R	P	T	V	A	G	I	F
Esccol	5.83	10.65	5.54	4.43	5.41	7.1	9.49	7.37	6	3.9
Shewone	6.47	10.97	4.64	4.06	5.37	6.76	9.42	6.79	6.03	3.97
Vibcho	6.33	10.9	4.95	4.02	5.19	7.07	9.15	6.68	6.04	4.08
Psying	4.82	10.99	4.08	3.71	5.49	6.57	8.49	6.68	7.53	4.47

	Y	C	H	Q	N	K	D	E	W	M

Esccol	2.85	1.17	2.27	4.43	3.95	4.41	5.14	5.75	1.53	2.8
Shewone	3.05	1.09	2.33	4.93	4.12	5.14	5.29	5.74	1.28	2.54
Vibcho	2.96	1.05	2.4	5.17	3.9	4.93	5.02	6.2	1.32	2.63
Psying	3.16	1.11	2.13	5.98	4.92	6.25	5.47	4.54	1.16	2.45

*Esccol = *Escherichia coli *MG1655, Shewone = *Shewanella oneidensis *MR-1, Vibcho = *Vibrio cholerae*, Psying = *Psychromonas ingrahamii *37

However, like the genome, the codons for these residues are GC rich, a factor that must be taken into account. Codon usage for *P. ingrahamii *is shown in Table 2 for all CDSs (coding DNA sequences) and for the highly expressed genes *tuf, tsf, fus*A+*RP *genes (P. Sharp, personal communication). Thus when amino acid content is examined as a function of GC3s, correcting for GC content at synonymously variable third positions, the overall amino acid composition of *P. ingrahamii *was found not to be remarkable. At the value of 34.2 determined for *P. ingrahamii*, amino acid contents fall on the curves generated for GC3s dependency in 80 other organisms (P. Sharp, personal communication) (data not shown) [9].

**Table 2:** Number of codons in highly expressed and in all genes of Psychromonas ingrahamii

		High	All			High	All			High	All			High	All
Phe	UUU	107	41275	Ser	UCU	139	14451	Tyr	UAU	47	25785	Cys	UGU	35	8165
Phe	UUC	103	10226	Ser	UCC	3	7455	Tyr	UAC	86	10612	Cys	UGC	5	4681
Leu	UUA	128	50155	Ser	UCA	73	16739	Ter	UAA	32	2366	Ter	UGA	1	571
Leu	UUG	44	17564	Ser	UCG	16	7210	Ter	UAG	7	608	Trp	UGG	31	13405
															
Leu	CUU	150	20860	Pro	CCU	98	13581	His	CAU	56	17366	Arg	CGU	408	17987
Leu	CUC	9	7631	Pro	CCC	5	9132	His	CAC	67	7202	Arg	CGC	74	11043
Leu	CUA	87	9170	Pro	CCA	110	10292	Gln	CAA	167	29958	Arg	CGA	12	5132
Leu	CUG	52	21318	Pro	CCG	32	9734	Gln	CAG	50	22366	Arg	CGG		3939
															
Ile	AUU	180	48789	Thr	ACU	189	17262	Asn	AAU	91	39859	Ser	AGU	48	19260
Ile	AUC	208	20430	Thr	ACC	31	19451	Asn	AAC	128	16887	Ser	AGC	60	14213
Ile	AUA	22	17588	Thr	ACA	113	16391	Lys	AAA	452	57148	Arg	AGA	14	6404
Met	AUG	188	28244	Thr	ACG	41	10226	Lys	AAG	134	14939	Arg	AGG	0	2583
Val	GUU	347	29210	Ala	GCU	328	26162	Asp	GAU	228	49174	Gly	GGU	412	33290
															
Val	GUC	32	13155	Ala	GCC	41	21828	Asp	GAC	83	13878	Gly	GGC	119	20816
Val	GUA	198	15326	Ala	GCA	273	30437	Glu	GAA	354	47661	Gly	GGA	33	12381
Val	GUG	52	18068	Ala	GCG	62	19459	Glu	GAG	91	21337	Gly	GGG	6	10497

#### Correspondence analysis (CA)

Lobry and Chessel [10] examined datasets of amino acid content and codon usage in thermophilic and mesophic bacteria and found evidence that the amino acid composition of thermophilic proteins was under the control of a pressure at the nucleic acid level, not a selection at the protein level. An extended study [11] produced similar results of no connection. However, the authors identified the most discriminating codon as being AGG for arginine, present in many thermophiles, not in either mesophiles or a psychrophile. *P. ingrahamii *fits this observation in that it does not use the AGG codon (Table 2).

**Figure 2:**
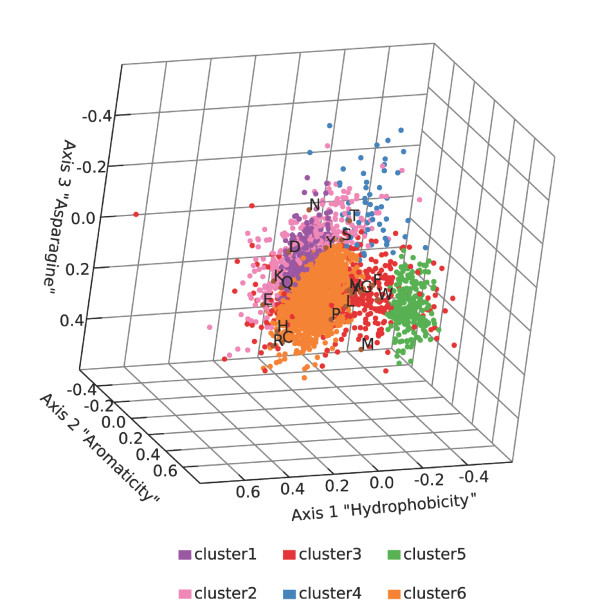
**Correspondence analysis of amino acid content of *P. ingrahamii *proteins**. Proteins over 100 residues were subjected to correspondence anlysis by amino acid content, clustered and plotted on first three most informative axes. Amino acid frequencies are superimposed. (See Methods).

We analyzed the amino acid compositions of all individual proteins of *P. ingrahamii *by CA [12] after trimming the first 10 and last 5 residues because there is a strong nucleotide bias at these positions. Data for each protein is presented in Additional file 1: "Ping Correspondence Analysis.xls". Clustering of groups of proteins with similar composition was performed using a bayesian approach as proposed by Bailly-Bechet *et al*. [13] Figure 2 presents a plot of the first three most informative axes: hydrophobicity, aromaticity and asparagine content.

The proteins fell into six classes whereas most bacteria contain at most five well separated clusters with bayesian clustering and at most four with dynamic clouds clustering [14]. Proteins of two bacterial proteomes examined to date fall into four clusters, *Aeropyrum pernix *and *Thermoplasma acidophilum *[14]. Subsequently it was seen that psychrophilic *P. haloplanktis *has 5 clusters [7], exceeded now by *P. ingrahamii*, a more extreme psychrophile, which resolves into six. In *P. ingrahamii*, the bulk of the proteins fall into three clusters (clusters 1, 2, 6). The integral inner membrane proteins (IIMPs) fall largely into clusters 3 and 5 (Figure 2). In other bacteria the IIMPs are distinctly separate from the bulk of other proteins [14]. In *P. ingrahamii*, however, separation is poor and there is some continuity between the IIMPs and the bulk proteins. Evidently the property of hydrophobicity is not distributed in psychrophile proteins as in mesophiles.

Not seen in any other bacteria viewed to date by CA is the sixth group, cluster 4. This group is of 57 proteins characterized by an excess of threonine that forms a bleb protruding from one of the core protein clusters. Examining the annotations of these proteins reveals that almost half are hypothetical proteins with no homologs in other bacteria examined at threshold Pam 150. It is tempting to suggest this group of proteins could be involved in facilitating low temperature growth.

As to amino acid content, one sees that asparagine occurs more frequently than expected for a random distribution, and on the other hand that cysteine, methionine, arginine and histidine are relatively rare (Figure 2). This same asparagine-driven bias has been seen in other psychrophiles, notably *P. haloplanktis *[7]. Deamidation via cyclization into aspartate threatens integrity of asparagine, a process which is sensitive to higher temperature, providing a rationale for an asparagine excess in psychrophiles. The corresponding lower amounts of cysteine, methionine, arginine and histidine can be understood as decreasing proportions of these oxygen-sensitive residues. Oxygen concentrations are higher in the liquid medium at low temperatures. Although similar composition gradients were seen in another psychrophile, *P. haloplanktis*, they are stronger in *P. ingrahamii*, perhaps correlated with its lower growth temperatures.

Thus, the main features that emerged from the CA of *P. ingrahamii *protein compositons (Figure 2) are that (1) there are more classes of proteins than have been seen in other bacteria, (2) one of these classes, cluster 4, has a high proportion of "orphan" hypothetical proteins, (3) IIMPs merge into bulk proteins rather than occupying a separate space, possibly due to IIMPs having lower hydrophobic character, and (4) one notes the strong opposition between asparagine, sensitive to heat, and the amino acids methionine, arginine, cysteine and histidine, sensitive to oxygen.

### Annotation

Of the 3545 genes for proteins of length 83 or more, 41 are fusions of two genes which in other organisms are separate and independent. They are distributed as 21 fused enzymes, 9 fused regulator components, 4 fused ABC transporter components, 4 fused phosphotransferase system (PTS) components and 3 mixed enzyme-regulator combinations. Fused genes can cause problems with annotation based on results of sequence similarity algorithms. To avoid such problems, we split fused genes for purposes of sequence comparisons in order to be able to identify orthologs of both parts independently.

The first round of annotation was carried out at Oak Ridge National Laboratory, posted for public access August 2006 [15]. We have here supplemented this data with manual analysis using the dynamic search programs of the Darwin system [16]. In this system after first approximations of sequence similarities, amino acid substitution tables are recalculated appropriate to degree of similarity and to the codon usage, and then pairwise alignments are produced. Degree of similarity between two sequences is expressed as percent identity and as Pam values [The Pam score (point accepted mutations) is an inverse measure of sequence differences] [17].

We first processed the *P. ingrahamii *protein sequences in relation to the then-completed 111 bacterial genomes. We extracted from this data the match with best (lowest) Pam score for each *P. ingrahamii *protein. The descriptions of the orthologs were retrieved from RefSeq and/or Genbank at the National Center for Biotechnology Information (NCBI) Web site [18]. The results allowed us to add some predicted protein products to the initial JGI annotation results.

Since the set of 111 bacterial genomes we first used was not balanced in respect to types of bacteria, we also identified orthologs in a reference set of 53 genomic sequences of organisms which were chosen to span the breadth of bacterial species. To include other sequenced psychrophiles, the set includes two additional marine species, *C. psychrerythraea *34 H and *I. loihiensis*. *C. psychrerythraea *34 H grows over the range -1C to 10C [5]; *I. loihiensis *has a broad temperature range from 4C to 46C [6].

Annotations and function information are attached in Additional file 2: "Ping Annotations 2.xls". The rank order of similar sequences and number of "best hits" are shown in Table 3.

**Table 3:** Organisms with similarity to greatest number of *P. ingrahamii *proteins*

Organism	Number of "Best Hits"
*Vibrio cholerae*	697
*Shewanella oneidensis*	539
*Colwellia psychrerythraea*	499
*Escherichia coli*	285
*Idiomarina loihiensis*	143
*Pseudomonas aeruginosa*	137

* sizes greater than 83 residues

Surprisingly, the organism with greatest similarity is *V. cholerae*. It is surprising because neither is *V. cholerae *one of the psychrophiles nor is it any member of the Order *Alteromonadale*s. *P. ingrahamii *is a member of the Family *Alteromonadacae *in the Order *Alteromondales*. *Psychromonas *is related to other members of this family such as *Shewanella*, *Colwellia *and *Idiomarina *[20]. Like some other members of these families, *P. ingrahamii *is a marine organism and has gas vesicles. We found there are extensive similarities with proteins of other Alteromonads as expected, but there are even more similarities with *V. cholerae*, even though the the Order *Vibrionales *and Family *Vibrionaceae *are separate from the *Alteromonadales *[19]. This observation may be explained by the selected conservation of genes from a common ancestor between these two genera and their loss by their closest relatives or it may indicate confusion due to unexpected horizontal gene transfer of 16S rDNA within these lineages.

In manually curating, we used practices aimed both at discovery and at caution. Whenever a "best match" was annotated as a hypothetical protein, yielding no information, we looked to the next best match. Sometimes the next best match was a very good one which provided useful information based on the annotated gene product of that ortholog.

It is widely appreciated in the genomics community that annotation by transfer of stipulated annotations from other organisms becomes ever more problematic at lower levels of similarity and as the number of sequential steps of sequence matches increases between the query and an experimentally demonstrated gene product. We were careful to be conservative in attributing a gene product if the match was not very close. When Pam scores were low (excellent match), we transferred directly the annotation of the match; but when in a middle range, 75–125, we sometimes generalized, removing specificity (i.e. we stipulated "an aminotransferase" instead of "aspartate aminotransferase"); and when Pam scores were high, over 125, we often used the word "predicted" to indicate that the assignment was based on a less rigorous threshold than for other assignments.

When formulating words of description of the gene products, we adopted another practice. We made an effort to name similar products similarly. We have tried to standardize product descriptions to some extent in order to make information on like proteins easier to find, thus making a list of alphabetized product names useful.

#### Assignments

From the above two sets of ortholog matches combined with the work of the JGI scientists, we constructed a table of *P. ingrahamii *gene numbers and corresponding best-guess annotated gene products available in Additional file 2: "Ping Annotations 2". Included in the table are the gene number, the type of gene product (enzyme, regulator, etc) and the name of the protein. (Gene number throughout this report is the number of the locus_tag which, as submitted December 2006 to Genbank, CP000510.1, has the prefix "Ping"). Altogether 217 proteins originally characterized as unknown gained an annotation by the manual process, some suggestive, others substantive.

#### Kinds of proteins

Table 4 gives classification of all proteins of *P. ingrahamii *by imputed function. The distribution of sizes of classes is similar to that for *Escherichia coli *K-12 [20] with enzymes being the largest class followed by transporters, then regulators, the rest divided into smaller categories.

**Table 4:** Distribution of functional types among all *P. ingrahamii *proteins*

Type of function	Number
Enzymes	1317
Unknown	761
Transporters	443
Regulators	252
Domains known	220
Factors	203
Structural	122
Horizontal	81
Carriers	47
Membrane	38
Cell process	38
Lipoproteins	21

Total	3545

* sizes greater than 83 residues

#### Horizontal transfer

Eighty-one genes were identifiable as currently known horizontally transferrable loci such as transposases of insertion sequences and integrases of phages. Thus clearly horizontal exchange of mobile elements into the chromosome has taken place. Among the bacteria tested, most such loci of *P. ingrahamii *matched elements of *Shewanella oneidensis *MR1. As is the case with so many genome sequences, we cannot know with currently available information how much of the genome of *P. ingrahamii *was formed by horizontal transfer and how much vertically inherited.

#### RNA

There are 10 ribosomal RNA clusters containing 5S, 16S, 23S RNAs (see Additional file 2). The relatively high number could reflect the need for high capacity of translation at cold temperatures and an ability to adapt quickly to changing conditions of nutrient availability [21]. 86 tRNA genes support translation.

#### Gene clusters

There are 100 contiguous clusters of two or more related genes such as subunits of an enzyme or pathway-related proteins. The largest cluster, for septum formation and peptidogylcan synthesis enzymes, comprises 14 genes. These are genes 1140 through 1153. Altogether 850, or 25% of CDSs, reside in clusters related by function.

#### Paralogous groups

Enumeration of sets of paralogs within a genome requires definition of degree of relatedness. In the initial analysis done at Oak Ridge National Laboratory, 1799 proteins were identified as belonging to 510 paralogous clusters. Using Darwin analysis [22] and a more conservative threshold of relatedness (Pam =< 175), 965 proteins were identified as grouped into 273 paralogous families of sizes ranging from two to 66. This threshold is comparable to that used to enumerate *E. coli *paralogs [23] and permits comparison. Functions of paralogous groups of size 7 or above are shown in Table 5. Just as had been found previously for *E. coli*, the largest paralogous groups are transporters and regulators. Even though enzymes are present in the genome in the largest numbers, they fall into smaller, more differentiated families. For transporters and also regulators, evidently a limited number of mechanisms have evolved for these functions, creating large groups of similar proteins of either transporters or regulators that differ in specificity but not in mechanism of action. Enzymes are more diverse, use a larger variety of mechanisms, thus they fall into a larger number of smaller groups of similar proteins.

**Table 5:** Distribution of functional types among largest *P. ingrahamii *paralogous groups

Group size	Protein function
66	ATP-binding subunits of ABC transporters
51	Cyclic-diGMP regulation, diguanylate cyclases
24	Transcriptional regulators, LysR type
23	Substrate-binding subunits of ABC transporters
15	Two-component response regulators
15	Transcriptional regulators, LacI type
12	Peptide-binding subunits of ABC transporters
11	ATP-dependent RNA helicases
11	Short-chain alcohol dehydrogenase family
10	Tripartite C4-dicarboxylate transporter, DctM-type subunits
9	Unknown
9	Fused ATP-binding/substrate-binding subunits of ABC transporters
9	IS4 transposases
8	Two-component sensor histidine kinases
7	IS30 transposases
7	Aldehyde dehydrogenases
7	Oxidoreductases
7	Tripartite C4-dicarboxylate transporter, DctQ-type subunits
7	Crotonase-like epimerase/dehydratases
7	Extracellular amino acid-binding subunits of ABC transporters
7	Aminotransferases

#### Intermediary metabolism

Almost half of all enzymes annotated in *P. ingrahamii *(634/1317) were identified as enzymes of small molecule metabolism. Not all enzymes of every pathway were found, not unexpected since some orthologs may not reach the threshold of similarity employed, nevertheless there is strong evidence for standard pathways of intermediary metabolism being present.

*P. ingrahamii *is a facultative anaerobe capable of both respiratory and fermentative metabolism [24]. In agreement we find in its enzyme sequences that pathways are present for fermentation, glycolysis, pentose phosphate pathway, the TCA cycle and gluconeogenesis. For respiration, electron transfer agents are present such as iron-sulfur centers, flavodoxin and flavoproteins, ubiquinone, and cytochromes; for anaerobic respiration, sequences of reductases for fumarate, nitrate, nitrite and sulfite are present (Table 6). Nitrate reduction has been observed experimentally [24]. In addition there are 10 members of the short-chain oxidoreductase family and 8 oxidoreductases identified by domain. Any one of these could be either a primary dehydrogenase or a terminal reductase not yet characterized (Table 7). The varieties of oxidants that appear to be used by *P. ingrahamii *suggest that its capabilities in this regard are comparable to that of *Shewanella *species.

**Table 6:** Genes and enzymes of glucose and energy metabolism

Pathway	Gene	Enzyme
Glycolysis		
	591	6-phosphofructokinase
	1316	6-phosphofructokinase
	669	enolase
	3617	fructose-1,6-bisphosphatase, class II
	2682	fructose-bisphosphate aldolase
	372	fructose-bisphosphate aldolase, class II
	2359	glucokinase
	2382	glucokinase
	2932	glucokinase, thermoresistant
	324	glucose-6-phosphate isomerase
	2004	glyceraldehyde-3-phosphate dehydrogenase,
	2367	glyceraldehyde-3-phosphate dehydrogenase,
	3636	glyceraldehyde-3-phosphate dehydrogenase,
	879	phosphoglucomutase
	769	phosphoglucomutase
	371	phosphoglycerate kinase
	249	phosphoglycerate mutase
	2320	phosphoglycerate mutase
	3211	phosphoglycerate mutase, cofactor-independent
	2361	pyruvate kinase
	2879	pyruvate kinase
	2199	triose-phosphate isomerase
		
Metabolic connections		
	3617	fructose-1,6-bisphosphatase, class II
	93	phosphoenolpyruvate carboxykinase
	537	malic enzyme
	304	isocitrate lyase and phosphorylmutase
	303	malate synthase
		
Pentose pathway		
	2752	6-phosphogluconate dehydrogenase, decarboxylating
	2937	6-phosphogluconate dehydrogenase, decarboxylating
	2753	6-phosphogluconolactonase
	167	ribulose-phosphate 3-epimerase
	2754	glucose-6-phosphate 1-dehydrogenase
	3554	ribose 5-phosphate isomerase
	601	ribose 5-phosphate isomerase
	2054	transaldolase
	86	transaldolase B
	339	transketolase
	3086	glucose dehydrogenase
	2936	gluconate kinase
		
Pyruvate dehydrogenase		
	2782	pyruvate dehydrogenase complex, E1 beta subunit
	3602	pyruvate dehydrogenase complex, E1 beta subunit
	3601	pyruvate dehydrogenase complex, E1 acetate transfer subunit
	3603	pyruvate dehydrogenase complex, E2 subunit
	2779	dihydrolipoamide dehydrogenase E3 subunit
	2780	dihydrolipoamide dehydrogenase E3 subunit
	2925	dihydrolipoamide dehydrogenase E3 subunit
		
Tricarboxylic acid cycle		
	2927	2-oxo-acid dehydrogenase E1 subunit
	2252	2-oxoglutarate dehydrogenase, E1 subunit
	2926	2-oxoglutarate dehydrogenase E2 subunit
	2251	2-oxoglutarate dehydrogenase, E2 subunit
	2899	aconitate hydratase
	2120	aconitate hydratase 1
	800	adenylyl-sulfate kinase
	2257	citrate synthase I
	2617	citrate synthase I
	1738	fumarate hydratase
	1977	fumarate hydratase
	983	isocitrate dehydrogenase, NADP-dependent
	297	malate dehydrogenase, NAD-dependent
	3376	oxaloacetate decarboxylase alpha subunit
	3375	oxaloacetate decarboxylase, beta subunit
	2253	succinate dehydrogenase catalytic subunit SdhB
	2254	succinate dehydrogenase, flavoprotein subunit SdhA
	2256	succinate dehydrogenase, cytochrome b-binding subunit sdhC
	2255	succinate dehydrogenase, cytochrome b-binding subunit sdhD
	2249	succinyl-CoA synthetase, alpha subunit
	2250	succinyl-CoA synthetase, beta subunit
		
Anaerobic respiration		
	3279	fumarate reductase iron-sulfur subunit
	3281	fumarate reductase, D subunit
	3278	fumarate reductase, flavoprotein subunit
	3280	fumarate reductase, subunit C
	2175	nitrate reductase accessory periplasmic protein NapD
	2172	nitrate reductase periplasmic cytochrome c-type protein NapC
	2173	nitrate reductase periplasmic cytochrome c-type subunit NapB
	2174	nitrate reductase, periplasmic large subunit
	1024	nitrite reductase [NAD(P)H], large subunit
	1023	nitrite reductase [NAD(P)H], small subunit
	3435	sulfite reductase (NADPH) hemoprotein, beta-component
	3434	sulfite reductase [NADPH] flavoprotein, alpha chain
	3436	phosphoadenosine phosphosulfate reductase (PAPS reductase)
		
Oxidoreductases of unknown substrate		
	45	short-chain dehydrogenase/reductase SDR
	223	short-chain dehydrogenase/reductase SDR
	951	short-chain dehydrogenase/reductase SDR
	989	short-chain dehydrogenase/reductase SDR
	1000	short-chain dehydrogenase/reductase SDR
	1973	short-chain dehydrogenase/reductase SDR
	2106	short-chain dehydrogenase/reductase SDR
	2109	short-chain dehydrogenase/reductase SDR
	2778	short-chain dehydrogenase/reductase SDR
	3154	short-chain dehydrogenase/reductase SDR
	272	oxidoreductase FAD/NAD(P)-binding domain protein
	3187	oxidoreductase FAD/NAD(P)-binding domain protein
	2122	oxidoreductase alpha (molybdopterin) subunit
	1244	oxidoreductase domain protein
	1809	oxidoreductase domain protein
	2666	oxidoreductase domain protein
	3535	oxidoreductase domain protein
	576	oxidoreductase, molybdopterin binding
		
Fermentation indications		
	91	fermentative D-lactate dehydrogenase
	2123	formate dehydrogenase, subunit FdhD
	1217	hydrogenase, NADP-reducing subunit C
		many alcohol dehydrogenases

**Table 7:** Metabolism of fatty acids

Pathway	Gene	Enzyme
Degradation	3600	acetyl-CoA synthetase
	2603	acyl-CoA dehydrogenase domain protein
	1208	enoyl-CoA hydratase/isomerase
	2604	fused 3-hydroxyacyl-CoA dehydrogenase, NAD-binding and enoyl-CoA hydratase/isomerase
	2401	acyl-CoA thiolase (acetyl-CoA transferase)
		
Synthesis	1090	acyl carrier protein
	1088	malonyl CoA-acyl carrier protein transacylase
	1995	3-oxoacyl-(acyl-carrier-protein) synthase I
	1087	3-oxoacyl-(acyl-carrier-protein) synthase III
	1997	3-oxoacyl-[acyl-carrier-protein) synthase III
	1091	beta-ketoacyl synthase
	1982	beta-hydroxyacyl-(acyl-carrier-protein) dehydratase
	1089	3-oxoacyl-(acyl-carrier-protein) reductase
	188	lauroyl (or palmitoleoyl)-ACP acyltransferase
	2965	(3R)-hydroxymyristoyl-ACP dehydratase
		
Unsaturation	1684	polyunsaturated fatty acid synthase
	1685	polyunsaturated fatty acid synthase
	1686	polyunsaturated fatty acid synthase
	1687	polyunsaturated fatty acid synthase

Enzymes are present for pathways of utilization of both carbohydrates and amino acids as carbon and energy sources. Glycerol was the carbon source provided in the medium for the below-freezing temperature growth experiments [8]. In agreement, genes for glycerol uptake (genes 3166, 3169), glycerol kinase (3168) and dehydrogenase (3207) are present.

Carbon sources besides glucose that were found experimentally to be utilized by *P. ingrahamii *[24] were compared with the list of enzyme orthologs. One finds in *P. ingrahamii *genes for enzymes for utilization of fructose (971, 3552), galactose (2016, 2017), mannitol (89), N-acetylglucosamine (488–490), ribose (344) and sucrose (974), in agreement with experimental findings. We also found genetic evidence for the capability to utilize lactose (2019) and glucuronate (130,131, 132 and 136).

However comparing to *E. coli *enzymes, many of the sequences of enzymes for utilization of other carbohydrates are not present in *P. ingrahamii*. These include enzymes for utilization of arabinose, xylose, sorbitol or galactitol. Again the absence is in agreement with experimental observations [24]. Also missing are orthologs of *E. coli *enzymes for utilization of tagatose, fucose, rhamnose, glucarate, galactarate, altronate or idonate. These carbon sources have not yet been tested in culture. This picture is similar to the one found in *S. oneidensis*, a relatively poor capacity to utilize a variety of monomeric carbohydrates [25].

Although capability for utilization of carbohydrates is restricted, by contrast *P. ingrahamii *does have sequences for enzymes for utilization of most amino acids, and many of these have been verified experimentally. Transaminases convert to the corresponding keto acids, decarboxylases to amines. The amino group can be removed with dehydratases or lyases.

#### Fatty acids, breakdown

The four major enzymes of degradation of fatty acids are present, capable of generating acetyl-CoA for general metabolism (Table 7).

#### Fatty acids, biosynthesis

It has long been known that proteobacteria at low temperatures adjust fatty acid composition to the more flexible unsaturated and/or branched types. *P. ingrahamii *has genes for enzymes of fatty acid biosynthesis (Table 7). Close similarity is found for all *E. coli *enzymes starting with malonyl-CoA, proceeding via malonyl-ACP through the fatty acid biosynthesis cycle, each cycle adding 2-carbon moieties. At the 10-carbon level, the pathway of unsaturated fatty acids commences. Orthologs for all *E. coli *enzymes of the unsaturation pathway are present except for the last enzyme, FabI. Final steps for synthesis of unsaturated fatty acids by *P. ingrahamii *must differ from those in *E. coli*. The principal fatty acids that were detected in the organism experimentally are the saturated 16:0 (18.7%) and the singly unsaturated 16:1ω7c (67.0%) [24].

*P. ingrahamii *has sequences of the four subunits of a polyunsaturase closely similar to the polyunsaturase in psychrophile *C. psychrerythraea *34H (Table 8). It is reasonable to suppose that at the very low temperatures of the environment *P. ingrahamii *might require fatty acids with more than one unsaturated bond. Also branched chain fatty acids are often found at cold temperatures. Based on enzyme sequences, precursors for synthesis of the branched-chain fatty acids could be generated as intermediates in breakdown of the amino acids leucine, isoleucine and valine. However, neither polyunsaturated nor branched chain fatty acids were detected in *P. ingrahamii *cultures grown at about 6 to 8°C (i.e. refrigerator) conditions [25]. Perhaps the polyunsaturated fatty acids are produced at lower temperatures or were not detected by the fatty acid analysis procedure used.

**Table 8:** Enzymes of macromolecule hydrolysis

Macromolecule	Gene	Enzyme
Peptides, protein	3027	D-alanyl-D-alanine carboxypeptidase, serine-type
	3293	D-alanyl-D-alanine carboxypeptidase/D-alanyl-D-alanine-endopeptidase
	314	O-sialoglycoprotein endopeptidase
	3325	prepilin peptidase type 4
	894	aminoacyl-histidine dipeptidase
	1331	aminopeptidase
	2545	aminopeptidase M24
	2344	aminopeptidase N
	1777	aspartyl aminopeptidase
	1034	carboxypeptidase thermostable
	2765	deacylase/carboxypeptidase family member, Zn-dependent
	409	dipeptidase
	395	leucyl aminopeptidase
	2026	metallopeptidase M24 family
	3003	methionine aminopeptidase, type I
	212	oligopeptidase A
	633	oligopeptidase B
	2690	peptidase
	1673	peptidase C1A, papain
	2671	peptidase C26
	268	peptidase M14, carboxypeptidase A
	2457	peptidase M14, carboxypeptidase A
	2444	peptidase M15B
	3480	peptidase M16 domain protein
	2127	peptidase M19, renal dipeptidase
	2301	peptidase M22, glycoprotease
	1544	peptidase M23B
	3212	peptidase M23B
	3225	peptidase M23B
	676	peptidase M23B, peptidoglycan-binding
	2784	peptidase M24
	1800	peptidase M48, Ste24p
	926	peptidase M48, Ste24p, Zn-dependent, TPR repeats
	686	peptidase M50
	3180	peptidase M50
	1350	peptidase M56, BlaR1
	2144	peptidase M6, immune inhibitor A
	2723	peptidase S1 and S6, chymotrypsin/Hap
	994	peptidase S16, LON domain protein
	925	peptidase S49
	1972	peptidase S49, N-terminal domain protein
	1301	peptidase U32
	2189	peptidase U32 family
	2460	peptidase dimerization domain protein
	410	peptidase domain protein
	478	prepilin peptidase dependent protein D
	606	proline aminopeptidase P II
	253	proline iminopeptidase
		
Polysaccharides	1954	alpha amylase, catalytic region
	3067	predicted glucoamylase I (alpha-1,4-glucan glucosidase)
	2381	alpha-D-1,4-glucosidase
	2383	dextran glucosidase
	554	glucan endo-1,3-beta-D-glucosidase
	893	glycogen debranching enzyme
	2363	glycogen debranching enzyme
	3070	glycogen debranching enzyme
	558	glycoside hydrolase family
	2014	glycoside hydrolase family
	2529	glycoside hydrolase family
	2841	glycoside hydrolase family
		
Murein	1782	gamma-D-glutamate-meso-diaminopimelate muropeptidase
	1075	lytic murein transglycosylase
	499	lytic murein transglycosylase, catalytic
	293	lytic transglycosylase, catalytic protein
	3319	lytic transglycosylase, catalytic protein
	293	lytic transglycosylase, catalytic protein
	367	lytic transglycosylase, catalytic protein
	3319	lytic transglycosylase, catalytic protein
		
Lipids	1779	esterase/lipase/thioesterase family protein
	1892	lipase, class 3
	2631	lipase-like
	2470	lipase/acylhydrolase family protein, GDSL-lilke
	258	phospholipase
	3493	phospholipase A(1)
	2455	phospholipase D/transphosphatidylase
	1334	phospholipase family, patatin-like protein
	1844	phospholipase family, patatin-like protein
	3290	predicted lysophospholipase
		
Nucleic acids	2776	5'-3' exonuclease
	201	ATP-dependent endonuclease of the OLD family
	501	DNA mismatch repair endonuclease mutH
	2451	HNH endonuclease
	317	TatD-related deoxyribonuclease
	807	TatD-related deoxyribonuclease
	716	crossover junction endodeoxyribonuclease
	1096	deoxyribonuclease of TatD family
	732	endonuclease III
	1020	endonuclease/exonuclease/phosphatase
	1518	endonuclease/exonuclease/phosphatase
	2456	endonuclease/exonuclease/phosphatase
	2694	endonuclease/exonuclease/phosphatase
	3327	endonuclease/exonuclease/phosphatase
	416	endoribonuclease L-PSP
	2129	endoribonuclease L-PSP
	2646	endoribonuclease L-PSP
	369	excinuclease ABC, A subunit
	2085	excinuclease ABC, A subunit
	1082	excinuclease ABC, B subunit
	1193	excinuclease ABC, C subunit
	2436	exodeoxyribonuclease I
	1319	exodeoxyribonuclease III
	1460	exodeoxyribonuclease V, alpha subunit
	1459	exodeoxyribonuclease V, beta subunit
	1458	exodeoxyribonuclease V, gamma subunit
	2951	exodeoxyribonuclease VII, large subunit
	2238	exodeoxyribonuclease VII, small subunit
	1303	exonuclease SMC domain protein
	1302	exonuclease SbcCD, D subunit
	2586	exonuclease of the beta-lactamase domain protein
	2270	exoribonuclease II
	2580	extracellular deoxyribonuclease
	53	formamidopyrimidine-DNA glycosylase
	2029	predicted endoribonuclease L-PSP
	2233	predicted exonuclease
	3302	single-stranded-DNA-specific exonuclease
	1283	uracil-DNA glycosylase
	1819	predicted ribonuclease BN
	3482	predicted ribonuclease BN
	1668	ribonuclease D
	496	ribonuclease H
	2962	ribonuclease HII
	640	ribonuclease III
	3609	ribonuclease P protein component
	3479	ribonuclease PH
	3417	ribonuclease R
	2450	ribonuclease T
	1126	ribonuclease, Rne/Rng family
	2208	ribonuclease, Rne/Rng family
	2214	tRNA-guanine transglycosylase
	2567	nuclease (SNase domain protein)
	2838	nuclease (SNase domain protein)
	265	exonuclease, RNase T and DNA polymerase III
	968	exonuclease, RNase T and DNA polymerase III
	3335	exonuclease, RNase T and DNA polymerase III

Interestingly, two polyunsaturated fatty acids, 20:5 (eicosapentaenoic acid) and 22:6 (docosahexaenoic acid), have been reported from two other species of *Psychromonas*, *P. kaikoae *and *P. marina*[25].

#### Glycogen storage

There are genes for 6 glucose-1-phosphate adenyltransferases (299, 1296, 2063, 3033,3034 and 3464) any one of which could serve for the first step in synthesis of glycogen, and there are 2 glycogen/starch synthases (2348 and 3035). One or more of the 15 glycosyl transferases could be involved in synthetic reactions.

#### Digestion of macromolecules

Like other marine organisms, *P. ingrahamii *appears to have the capability of utilizing macromolecules in the environment for nutrition and energy. *P. ingrahamii *has genes for a relatively large number of 48 peptidases and proteases (Table 8). Some of these are no doubt required for internal turnover, but some seem likely to be exported out of the cell in order to hydrolyze environmental proteins, thus providing small molecular weight nutrients for uptake. *P. ingrahamii *has a complete general secretion system capable of excreting such degradative enzymes to the environment. To take up the digestion products of proteolysis, there are ABC-type transporters for peptides, many for amino acids. Not consistent with this prediction is the experimental observation that gelatin is not hydrolysed [24].

Storage glycogen as well as external polysaccharides including starch could be hydrozysed for production of sugars to supply energy. For utilization of some polysaccharides there are amylases, glucosidases, debranching enzymes, and glycosyl hydrolases (Table 8), some of which may be intracellular and others extracellular. There are 7 lytic transglycosylases which, in cleaving peptidoglycan links could be involved in modellingof the cell or could break up environmental cell wall fragments. Capability to hydrolyse fats also exists as there are 3 lipases, 5 phospholipases and a lyso-lipase. There are many enzymes hydrolyzing nucleic acids with different specificities and functions, many with vital internal metabolic roles. In addition, some may be used to hydrolyze external nucleic acid debris (Table 8).

Other hydrolases are encoded in the genome whose physiological roles are not currently known, for example there are 11 HAD-superfamily hydrolases, 7 alpha/beta hydrolase fold proteins, 5 metal-dependent phosphohydrolases.

#### Chaperones and stress proteins

Multiple chaperone proteins are encoded in *P. ingrahamii*, suggesting that folding of proteins is an important process (Table 9). There are 4 proteins like DnaK, 4 like DnaJ, 3 GroEL monomers and 2 GroES monomers There are 12 peptidylprolyl isomerases (trigger factors that act at nascent polypeptide chains), and a ClpB protein disaggregating complex. One can speculate that the the role of the chaperones is to guide nascent polypeptides into functional three-dimensional configurations permitting activity at low temperatures. Future characterization of some of these chaperones could reveal what kinds of folding are required to retain protein function at sub-zero temperatures. Ferrer *et al*. [26] found that GroEL of *Oleispira antartica *RB8 functioned as a single ring of 7 units at cold temperatures, but as a double ring of 7 over 7 at warm temperatures. They pinpointed two residues as critical to the transition from double to single ring. However on inspection these residues do not occur at the comparable positions in *P. ingrahamii *GroEL proteins. Actions of *P. ingrahamii *chaperones at cold temperatures remain to be explored.

**Table 9:** Chaperones and stress proteins

Category	Gene numbers	Protein
Chaperones	917, 1232, 1233, 1328	DnaK-like
	918, 1039, 2621, 2499	DnaJ-like
	843, 2494	GroES
	844, 2493, 2791	GroEL
	919, 1049, 1080, 1469, 1619,	Peptidyl prolyl isomerases
	1856, 1917, 2185, 3116, 3199,	(trigger factors)
	3257, 3269	
	1040, 3623	ClpB disaggregator
		
Stress proteins	279, 755, 1097, 1881, 1953,	Cold Shock
	2158, 2543, 2698,2701, 3095,	
	3098, 3704	
	3, 95, 202, 956, 1039, 1051,	Heat shock
	1246, 1533, 1806, 1916, 2499,	
	2692	
	125, 930, 954, 955, 959, 1234,	Universal stress proteins
	2734	
	378, 1265, 1264, 1265, 1266,	Tellurite resistance
	2005, 2574, 2575	(anti_superoxide)

*P. ingrahamii *has genes for a variety of known types of stress proteins: There are 12 cold shock proteins, 9 heat shock proteins, 7 UspA-type stress proteins as well as 9 "tellurite resistance" proteins now known to protect against superoxide formation [27] (Table 9). Experimental work will be needed to determine which if any of these have functions directed specifically at living in cold temperatures and if there are other types of stress proteins or any other cold-associated functions among the open reading frames of unknown function.

#### Transporters

Compared to *E. coli*, *P. ingrahamii *has few transporters specific for sugars and sugar alcohols, but does have many transporters for amino acids (see Additional file 2). In this respect, it seems that *P. ingrahamii *is like *S. oneidensis *and other *Alteromondales *in a capacity to utilize environmental amino acids as carbon (and nitrogen) sources, contrasted to less capacity for sugars.

As to types of transporters, the ABC type of ATP-driven multisubunit transporter is most common in the *P. ingrahamii *genome, as is the case for other bacteria. Also, as for many other bacteria, conventional secondary transporters follow in frequency. However, *P. ingrahamii *differs from many in the fact that in there are 11 sets of the *dct*M, *dct*P and *dct*Q genes for the tripartite ATP-independent periplasmic transporter systems (TRAP) [28,29] (Table 10), more than are found in the three mesophiles whose whole genomes were compared (*E. coli*, *S. oneidensis *and *V. cholerae*). TRAP systems specialize in transport of C4-dicarboxylic organic acids such as fumarate, perhaps for anaerobic respiration purposes. The number of 3-gene TRAP systems in bacteria is variable. *E. coli *has one, *Pseudomonas aeruginosa *has 6 whereas *P. ingrahamii *has 11 of these three-protein systems. Recently 15 TRAP systems have been identified in *Sinorhizobium meliloti *1021 [30]. TRAP transporters use a proton motive force energized system, simpler and possibly more primitive than the ATP-utilizing ABC systems. A connection between the TRAP transporters and low temperature growth is not currently known.

**Table 10:** Tripartite ATP-independent C4-dicarboxylate transporters

DctM-like	DctQ-like	DctP-like
IIM* subunit	IIM* subunit	Periplasmic subunit

Gene	Gene	Gene

133	134	135
538	539	540
572	571	570
646	647	648
710	709	708
2033	2034	2035
2595	2595	2594
2935	2934	2933
3148	3149	3150
3544	3545	3546
3673	3674	3675

*IIM = integral inner membrane

#### Regulators

Many types of regulation mechanisms have been identified in *P. ingrahamii*: transcriptional activators and repressors, cyclic-AMP regulation, chemotaxis systems, two-component sensor-response regulators of several types including the twin-arginine translocation (Tat) pathway signal sequence domain proteins, also synthesis/breakdown of cyclic-diGMP signalling second messengers associated with GGDEF and/or EAL domains (see Additional file 2). There are 61 regulators of the cyclic-diGMP signalling second messenger in the genome, compared to 29 in *E. coli *K-12 MG1655. Cyclic-diGMP concentrations are controlled by either a diguanylate cyclase or a specific phosphodiesterase or both, together governing synthesis or hydrolysis of the cyclic-GMP. The types of genes and corresponding physiology regulated by cyclic diguanylate systems so far identified are motility, adhesion factors, fimbriae and biofilm formation.

Given the finding of a large number of cyclic-diGMP signalling systems, we might guess that *P. ingrahamii *lives within the matrix of a biofilm. Altogether 16 glycosyl transferases are encoded (by genes 326, 327, 328, 329, 331, 336, 440, 449, 454, 456, 457, 779, 792, 1794, 3458, and 3647), suggesting that polysaccharide, perhaps exopolysaccharide, is a major synthetic product. For export, many efflux proteins are present. An ortholog is present (1200) of the quorum sensing HapR (LuxR) regulator that is involved in controlling biofilm formation in *V. cholerae *[31,32]. An extracellular matrix, a major physiological feature of the related *S. oneidensis *[33], could well be important to life in the cold, providing stability and resilience to the population. Since *P. ingrahamii *lives in sea ice, it is possible that extracellular polysaccharide (EPS) may be part of the sea ice microbial community (SIMCO) biofilm although it should be noted that *P. ingrahamii *was isolated from the ice column above the major biofilm of the SIMCO.

Alternatively, the production of EPS may serve a role in sequestering water from ambient saltwater at lower temperatures or actually lowering the freezing point. In this regard it is interesting to note that in the -12°C growth experiments, none of the tubes froze after the first week of growth following inoculation suggesting that a product, possibly EPS, produced by the bacterium may have lowered the freezing point of the growth medium.

Regulation of expression of certain classes of genes is moderated by the sigma factors of the RNA polymerase holoenzyme. *P. ingrahamii *is well endowed, appearing to have genes for sigma factors 24 (RpoE), 32 (RpoH), 38 (RpoS), 54 (RpoN) and 70(RpoD) [gene numbers are, respectively, 65, 626, 677, (424, 712, 2892 and 3175 for sigma-54) and (310, 946 and 995 for sigma-70)]. RpoE and RpoH are both stress-responding factors. RpoS operates in stationary phase and also under stress. RpoN is concerned with nitrogen metabolism and in *V. cholerae *regulates flagellin gene transcription. RpoD for sigma 70 is the primary factor in *E. coli*.

#### Ribosomes

There are 58 ribosomal proteins annotated, 5 of which are duplicated, therefore 53 unique. Of these, 38 were found as orthologs with Pam values less than 40 among the two sets of genomes we examined. Organisms with closest matches were, in descending order, *V. cholerae*, *V. parahaemolyticus*, *S. oneidensis*, *H. influenzae *= *I. loihiensis*, *C. psychrerythraea*. Again, the close relationship of *P. ingrahamii *with Vibrio species is evident.

#### Osmotic stability

*P. ingrahamii *grows well over the range 1 to 12% NaCl [25]. To manage the potential for osmotic imbalance, the genes for enzymes to synthesize the osmolyte glycine betaine from choline are present (genes 2071, 2072), as well as a transporter to take up choline (gene 969) or, bypassing synthesis, specific ABC transport systems for uptake of glycine betaine are present, (genes 614–616, 2073–2075). This capability may explain how the organism is able to survive and perhaps grow in the salt pockets that are formed within the sea ice.

#### Motility

There is a large cluster of flagellar genes in *P. ingrahamii *in the region between gene numbers 3562 to 3598 and four copies of sigma factor rpoN which is known in *V. cholerae *to regulate flagellin genes. Yet the bacteria in culture have been observed to be non-motile [8]. There may be a defect in one of the essential flagellar proteins or in the expression or assembly processes. Alternatively, the organism may not always express flagella formation and motility. Interestingly, the original description of the genus indicates that other members of the genus are motile [34].

#### Gas vesicles

Two kinds of gas vesicles have been observed in the cell under culture (24). Genes orthologous to known gas vesicle genes are present in two clusters, the ranges 1248 to 1262 and 1748 and 1750. Although the two types can be expressed simultaneously in cells, they may be differentially expressed under preferential conditions in the environment.

#### Different kinds of enzyme orthologs in different bacterial relatives

Of the 53 bacteria chosen to represent the breadth of the currently characterized eubacterial world, the three bacteria bearing the largest number of protein sequences scored as "best matches" with *P. ingrahamii *proteins are *V. cholerae*, *S. oneidensis *and *C. psychrerythraea *34H (Table 3).

About 3/4 of the *P. ingrahamii *proteins annotated as enzymes, transporters or regulators have orthologs at the level of Pam <= 125 in either *V. cholerae, S. oneidensis *or *C. psychroerythraea*. By contrast, only 19% of *P. ingrahamii *CDSs of unknown function have orthologs in these bacteria. That the vast majority, 4/5, of *P. ingrahamii *CDSs of unknown function do not have good matches in the most closely related organisms suggests that many proteins in *P. ingrahamii *are qualitatively different. New, unique functions will not be revealed by annotations that depend on similarity to known proteins, but their existence suggests that further experimental study of this extremophile is worthwhile and could bring new biology to light.

Looking within the enzyme category, it is striking that most of the best hit homologs of enzymes of central metabolism are proteins of *V. cholerae*. This is particularly puzzling since *V. cholerae *is a mesophile and it is phylogenetically in a different Order and Family from *P. ingrahamii*. Could this be an example of massive horizontal transfer of genes from *Vibrio *to *Psychromonas*? It seems unlikely since the many genes for central metabolism are not in a few clusters similar to pathogenicity islands, rather they map throughout the chromosome, and the majority of the transposase-type genes have closest orthologs in *S. oneidensis*. Nevertheless, in spite of expectations, we have found that the sequences of enzymes of basic metabolism that function at low temperature are more similar to those of a mesophile than they are to those of another psychrophile, and even more surprising, a mesophile of a different taxonomic Order and Family. A feature that may suggest global genome reorganization and account for an apparent scrambling of otherwise conserved genes is that the Vibrios usually comprise two chromosomes, in contrast to *P. ingrahamii *which has only one.

Different from *V. cholerae*, one sees that enzyme homologs in *S. oneidensis *and *C. psychrerythraea *34H are largely the enzymes of peripheral and macromolecule metabolism, not the enzymes of central metabolism.

We narrowed our question, looking for a commonality in enzymes among the psychrophiles. We identified the 566 proteins in *C. psychrerythraea *and *I. hoiliensis *that have highest similarity to *P. ingrahamii *proteins at Pam values =< 125. In this set, there are few enzymes important to central metabolism; many more are involved in macromolecule synthesis and maintenance. There are enzymes dealing with nucleic acids: exo and endo-nucleases, RNA helicases, DNA polymerase and helicases, transcriptional and translational factors, and recombinases. Psychrophily may require differences in the proteins of nucleic acid metabolism since even though the salt content of the sea ice and the low GC content of the DNA (40.1%) should act to lower the melting point of double stranded polynucleotides. However, handling nucleic acid structures at such cold temperatures may also require differences in the interacting proteins. Similarly, transcription and translation factors of a particular kind may be required to enable the processes at low temperatures.

#### Unique unknown proteins

We identified a few small groups of proteins that could perform novel functions related to psychrophily, warranting further investigation in the future. A contiguous set of nine genes that could comprise an operon, gene numbers 3053 through 3061 have no orthologs in current databases with Pam value <150. Four of the proteins are paralogous, with similar sequences among themselves. As we are looking for functions in psychrophiles not found in other bacteria, experimental characterization of this group of proteins might be worthwhile.

There are 3 groups of genes that could be contiguous operons whose members reside either in cluster 4 of the Correspondence Analysis (*vide supra*), or are unknown in other bacteria to date, or both. These are clusters 1672 through 1676, 1960 through 1964, and 2315 through 2319. Could any or all of these relate to the ability to live and grow at -12C or lower temperatures? We identify these seemingly unique sets of proteins with the thought that when results of proteomic expression experiments on this organism are available, it might be useful to characterize the proteins and to know under what circumstances they are synthesized.

## Conclusion

The *P. ingrahamii *genome, although it is 25% smaller than the genome of *E. coli *K-12 MG1655 and has many more CDSs labeled hypothetical, is similar to *E. coli *in the distribution of functions among annotated proteins, with enzymes by far the largest category followed by transporters, then regulators. Other categories follow distantly. Similar to *E. coli*, the largest paralogous groups within the genome were transporters and regulators, the smaller ones enzymes. Although transporters and regulators are fewer in number than enzymes, they form larger paralogous groups. They belong to fewer families employing fewer mechanisms than is the case for enzymes which belong to smaller families of greater variety.

Unexpectedly, *P. ingrahami*i protein sequences were similar to more *V. cholerae *proteins than to proteins from *S. oneidensis *or *C. psychrerythraea*. This is in spite of the fact that *Vibri*o species belong to a different Family and Order than do Psychromonadaceae, Shewanellaceae and Collwelliaceae, and in spite of the fact that *C. psychrerythraea *is also a psychrophile.

In a comparison of gross properties of all the proteins of *P. ingrahamii *differences were not found from those of three mesophiles in several respects: distribution of lengths, total amino acid composition, codon usage when corrected for genomic GC content.

However, correspondence analysis (CA) of the amino acid content of the proteins showed that they cluster in ways that differ from most other bacteria, falling into more clusters that are not as well separated from one another as in other bacteria. This may be a consequence of an unusual distribution of hydrophobicity. One of the clusters is composed almost half of unidentified, unknown CDSs. *P. ingrahamii *contains proteins with relatively high asparagine content, and low content of amino acids potentially sensitive to the higher concentration of oxygn present in cold waters. These properties would seem to be appropriate for an extreme psychrophile.

As to metabolism, *P. ingrahamii *is a facultative anaerobe capable of both fermentation and respiration. In agreement, proteins similar to the corresponding metabolic pathways and proteins for synthesis and harvest of glycogen storage compound were identified. Most enzymes of central small molecule metabolism were most closely related to those of *V. cholerae*. Enzymes of macromolecular synthesis and maintenance are most closely related to those of *S. oneidensis *and *C. psychrerythraea*. There seems to be a preference of amino acids over sugars as sources of carbon and energy, perhaps harvested by extracellular degradation of environmental proteins by exported peptidases. To create a more flexible lipid layer adjusted to cold temperatures, sequences of a heteropolymeric polyunsaturase were found. However, the enzyme may not always be active as no polyunsaturated fatty acids were detected in culture. Perhaps, however, in nature they are expressed at temperatures lower than the lowest normally used for laboratory cultivation, 6–8°C.

In addition to transporters of types common to other proteobacteria were 11 three-component TRAP systems for C4-dicarboxylic acid transport. In addition to regulators of types common to other proteobacteria were 61 regulators of cyclic-diGMP second messengers, suggesting that biofilms and their regulation could be an important part of the life style of this psychrophile. Alternatively, *P. ingrahamii *may produce EPS to lower the freezing point in the surrounding environment, thereby making water available for low temperature growth.

Looking for particular proteins that might be unique to this psychrophile but are not yet in the genomic databases we searched, we have pointed out some sets of gene products that could be starting points for discovering new functions or new types of proteins. We noticed 9 contiguous genes that are all unknown hypotheticals, 4 of them sequence-related to one another. We also noted three apparent contiguous operons, members of which are unknown hypotheticals and/or members of the unique set of proteins in cluster 4. In future, we recommend that proteomic experiments should be used to explore the shift to cold temperature with a view of keeping an eye out for expression of any of these proteins, potentially part of the mechanisms of life at very cold temperatures.

## Methods

### Genome sequencing and finishing

The genome of *Psychromonas ingrahamii *was sequenced at the Joint Genome Institute Lawrence Laboratories, Walnut Creek, CA using a combination of (4 kb, 6.8 kb and 36 kb) DNA libraries. All general aspects of library construction and sequencing performed can be found on line [35]. Draft assemblies were based on 53473 total reads. All three libraries provided 11X coverage of the genome. For closing and finishing, the Phred/Phrap/Consed software package [36,37] was used for sequence assembly and quality assessment [38,39]. After the shotgun stage, 53473 reads were assembled with parallel phrap (High Performance Software, LLC). Possible mis-assemblies were corrected with Dupfinisher [41] or transposon bombing of bridging clones (Epicentre Biotechnologies, Madison, WI). Gaps between contigs were closed by editing in Consed, custom primer walks, or PCR amplification. A total of 952 primer walk reactions and 3 transposon bombs were necessary to close gaps, to resolve repetitive regions, and to raise the quality of the finished sequence. The completed genome sequences of *Psychromonas ingrahamii *contain 53,592 reads, achieving an average of 11-fold sequence coverage per base with an error rate less than 1 in 100,000. The sequence of *P. ingrahamii *can be accessed using the GenBank accession number CP000510.1.

### Annotation

Annotation was initially carried out at Oak Ridge National Laboratory using methods detailed in [41,42]. Further annotation and analysis of protein sequence similarities used the Darwin system (Data Analysis and Retrieval With Indexed Nucleotide/peptide sequence package), version 2.0, developed at the ETHZ in Zurich, Switzerland [16,22]. Pairwise sequence alignments and scores were generated using the AllAllDb program of Darwin. Maximum likelihood alignments are generated with an initial global alignment by dynamic programming (Smith and Waterman algorithm) followed by dynamic local alignments (Needleman and Wunsch algorithm). A single scoring matrix is used for these steps. After the initial alignment, the scoring matrix is adjusted to fit the approximate distance between each protein pair to produce the minimum Pam value. Pam units are defined as the numbers of point mutations (base pair differences) per 100 residues [17]. The Darwin system's ability to apply scoring matrices according to the distance between each protein pair ensures a data set of highly accurate similarity calculations for distantly as well as closely related protein pairs. The identification of distantly related proteins is valuable in finding divergent but related protein functions.

To extract homolog matches from initial data, we required that the alignments with *P. ingrahamii *proteins be at least 83 residues long [43], and that the alignment must represent over 40% of both proteins. Unless otherwise stated we required Pam scores to be 150 or less. To assemble groups of paralogs, the Pam threshold for pairs was raised to 250, and then pairs were grouped by a transitive process as previously described [23].

### Correspondence Analysis

The amino acid composition of the proteins of *P. ingrahamii *was analyzed using correspondence analysis (CA) [12-14] with the FactoMineR R package [44]. Each protein was truncated for the first 10 and last 5 amino acid residues and each is represented by its normalized aminoacid content. The representation is in a 19-dimension space where specific statistical distances are measured by the chi-square method. The 3 most informative dimensions are used to plot the position of each individual protein. The individual amino acid distributions are superimposed in this same space. The axes are numbered in order of the amount of information they carry. A bayseian method, as in Bailly-Bechet et al. [41], was used to cluster proteins of similar composition using the Mclust R package [45], and the best classification, which is associated with the largest BIC (Bayesian Information Criterion) value, was selected for further analysis.

Codon usage and selection bias were determined by methods of Sharp *et al*. [9].

## Authors' contributions

MR used Darwin-generated data for further annotation and analysis and wrote the paper. JTS made biological determinations and made significant contributions to writing the paper. AD and TZW carried out and interpreted the corresponence analysis. AD made significant contributions to writing the paper. JST and TSB closed and finalized the sequence. MLL and LJH carried out the initial annotation and submitted to GenBank.

## Supplementary Material

Additional file 1 **Ping Correspodence Analysis**. Correspondence analysis of amino acid content of *Psychromonas ingrahamii *proteins. Gene number, Cluster number, Product Click here for file

Additional file 2 **Ping Annotations 2**. Further annotation of *Psychromonas ingrahamii *proteins. Gene number, Gi identifier, predicted protein product, type of product Click here for file
